# Six-band terahertz metamaterial absorber based on the combination of multiple-order responses of metallic patches in a dual-layer stacked resonance structure

**DOI:** 10.1038/srep41373

**Published:** 2017-01-25

**Authors:** Ben-Xin Wang, Gui-Zhen Wang, Tian Sang, Ling-Ling Wang

**Affiliations:** 1 Jiangsu Provincial Research Center of Light Industrial Optoelectronic Engineering and Technology, School of Science, Jiangnan University, Wuxi 214122, China; 2 Library and Information Center, Hunan Traditional Chinese Medical College, Zhuzhou 412012, China; 3 School of Physics and Electronics, Hunan University, Changsha 410082, China

## Abstract

This paper reports on a numerical study of the six-band metamaterial absorber composed of two alternating stack of metallic-dielectric layers on top of a continuous metallic plane. Six obvious resonance peaks with high absorption performance (average larger than 99.37%) are realized. The first, third, fifth, and the second, fourth, sixth resonance absorption bands are attributed to the multiple-order responses (i.e., the 1-, 3- and 5-order responses) of the bottom- and top-layer of the structure, respectively, and thus the absorption mechanism of six-band absorber is due to the combination of two sets of the multiple-order resonances of these two layers. Besides, the size changes of the metallic layers have the ability to tune the frequencies of the six-band absorber. Employing the results, we also present a six-band polarization tunable absorber through varying the sizes of the structure in two orthogonal polarization directions. Moreover, nine-band terahertz absorber can be achieved by using a three-layer stacked structure. Simulation results indicate that the absorber possesses nine distinct resonance bands, and average absorptivities of them are larger than 94.03%. The six-band or nine-band absorbers obtained here have potential applications in many optoelectronic and engineering technology areas.

In recent years, metamaterial-based resonance devices have attracted considerable attention due to the fact that they have the ability to manipulate and control the incident electromagnetic (EM) wave at sub-wavelength dimensions. These devices are, but are not limited to modulators^1^, filters^2^, absorbers^3^, switches^4^, polarization conversions^5^ and electromagnetically induced transparency (or absorption)^6^,^7^. Among them, metamaterial perfect absorber (MPA)^8^,^9^,^10^ has garnered interest because they have many potential application prospects in optoelectronic related areas, including photovoltaics and solar cells, thermal radiation and imaging, materials detection, and so on. Therefore, since the MPA first presentation in the year of 2008^3^, the MPAs based on various resonance structures^11^,^12^,^13^,^14^,^15^,^16^,^17^,^18^,^19^,^20^,^21^,^22^,^23^,^24^,^25^,^26^ have remarkably progressed from microwave to optical. However, most of MPAs presented here have the common problems of narrow-band or single-band absorption, as a result of the strong EM response of the metamaterials. This kind of resonance characteristic greatly restricts the application prospects of the MPAs. In many application areas, hence, it is necessary to develop and design the multiple-band MPAs.

The multiple-band MPAs can be easily obtained by coplanar or stacked structures composed of several different dimensions of the metallic elements (or resonators)^27^,^28^,^29^,^30^,^31^,^32^,^33^,^34^,^35^,^36^,^37^,^38^,^39^,^40^,^41^,^42^,^43^,^44^,^45^,^46^,^47^,^48^,^49^,^50^,^51^,^52^,^53^. However, because the limitation of the number of the resonators or elements in the metamaterial structures, the research of the multiple-band MPAs is mainly concentrated in dual-band^27^,^28^,^29^,^30^,^31^,^32^,^33^,^34^, triple-band^35^,^36^,^37^,^38^,^39^,^40^,^41^,^42^ and quad-band absorption^43^,^44^,^45^,^46^,^47^,^48^,^49^,^50^,^51^,^52^,^53^. In other words, the number of the absorption peaks of the current MPAs is no more than four. More importantly, in these structures, we found that the design of these multiple-band MPAs is mainly based on the combined effect of the fundamental mode resonance (i.e., the 1-order response) of the metallic patterns. The investigation of the high-order responses of the metallic patterns is rarely reported. In this paper, we extend the analysis of MPAs by making full use of the high-order responses of the metallic patterns to achieve the multiple-band (including the six-band and even the nine-band) absorption responses.

Moreover, for the applications of the selective thermal emitter, and thermal imaging, multiple-band MPAs, especially which have more than four resonance peaks (for example six-band absorption) are in urgent need of development. Unfortunately, these kinds of multiple-band MPAs are seldom demonstrated. However, according to the strategy of the multiple-band MPAs that using the aggregation effect of the fundamental responses of the metallic patterns in coplanar or stacked layers, the design of the six-band MPA inevitably has complex resonance structure and time-consuming processing steps.

This paper presents the design of the six-band MPA based on multiple-order responses of metallic patterns at terahertz frequency. The demonstrated six-band MPA is composed of two alternating stack of metallic-dielectric layers on a metallic board. Numerical studies prove that the designed MPA possesses six distinct resonance bands, and the average absorption coefficients of them are greater than 99.37%. Each layer of resonance structure has three resonance modes (1-order, 3-order and 5-order responses), and thus the mechanism of the six-band absorption is attributed to the combination of two sets of three different resonance modes (1-order, 3-order and 5-order responses) of the two metallic layers. The mechanism of the presented multiple-band MPA is different from those in most references that only utilize the superposition of the fundamental mode response (i.e., 1-order resonance) of metallic patterns. Besides, the resonance frequencies of the six-band MPA can be tuned by varying the sizes of the metallic layers and the thicknesses of the dielectric layers. Using the results, we also investigate and study a polarization tunable six-band MPA through adjusting the sizes of the metallic layers in two perpendicular (or orthogonal) polarization directions. Furthermore, the number of the resonance bands or peaks can be further increased by stacking more metallic layers, for example, nine-band terahertz MPA is obtained by utilizing a three-layer stacked resonance structure. Six-band and even nine-band MPAs achieved here have many practical applications, such as thermal imaging and radiation, and the investigation and study of the multiple-band MPAs will facilitate the development of the metamaterial devices.

## Results and Discussion

**Figure 1:**
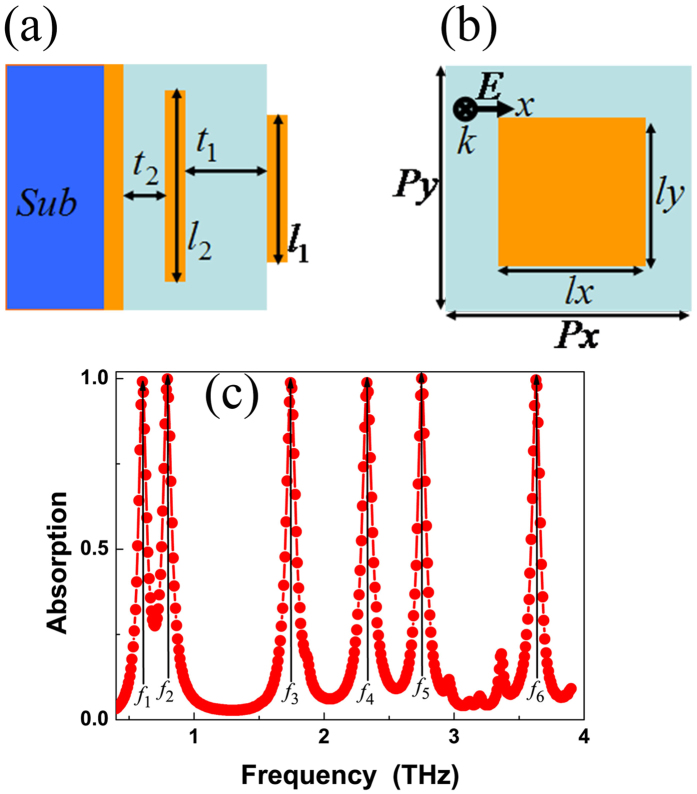
(**a**) and (**b**) show the side- and top-view of the six-band MPA, respectively, the parameters (in μm) of the MPA are: *l*_1_* = l*_1x_ = *l*_1y_ = 55, *l*_2_* = l*_2x_ = *l*_2y_ = 65, *t*_1_ = 6, *t*_2_ = 4.5, *P* = *Px* = *Py* = 75; (**c**) is the absorption spectrum of the designed six-band MPA.

Figure 1(a) and (b) show the side- and top-view of the six-band MPA, respectively. As shown in Fig. 1(a), the presented six-band MPA is a dual-layer stacked resonance structure, and the length of the top metallic square patch (*l*_1_) is smaller than that of the bottom square patch (*l*_2_). Detailed parameters (in μm) of the six-band MPA are: *l*_1_* = l*_1x_ = *l*_1y_ = 55, *l*_2_* = l*_2x_ = *l*_2y_ = 65, *t*_1_ = 6, *t*_2_ = 4.5, *P* = *Px* = *Py* = 75. The calculation results of the whole paper are carried out using FDTD Solutions, which is based on finite-difference time-domain method. The other geometrical parameters of the presented MPA and the computational model as well as the appropriate boundary conditions applied in this paper are shown in the Section of the Methods.

Figure 1(c) presents the simulated absorption spectra of the designed six-band MPA. As you can seen in Fig. 1(c), six obvious absorption peaks or bands at frequencies of 0.60 THz (*f*_1_), 0.80 THz (*f*_2_), 1.74 THz (*f*_3_), 2.33 THz (*f*_4_), 2.75 THz (*f*_5_), and 3.63 THz (*f*_6_) with high absorption coefficients of 99.10%, 99.90%, 98.77%, 98.82%, 99.99%, and 99.63% are observed, respectively. The bandwidths (full width half maximum) of the six resonance peaks from low- to high-frequency are 0.079 THz, 0.088 THz, 0.097 THz, 0.097 THz, 0.079 THz, and 0.079 THz, respectively. The *Q* (the resonance frequency with respect to the bandwidth) values of the modes *f*_1_, *f*_2_, *f*_3_, *f*_4_, *f*_5_, and *f*_6_ are 7.59, 9.09, 17.94, 24.02, 34.81, and 45.95, respectively. Meanwhile, the off-resonances (for example at 0.70 THz, 1.32 THz, 2.10 THz, 2.50 THz, 3.20 THz, and 3.80 THz) absorption coefficients of presented MPA are quite low, not exceeding 30%. These resonance characteristics indicate that the six absorption bands are apparently discernible and the perfect absorption with quite narrow bandwidth merely appears near the operating frequency.

**Figure 2:**
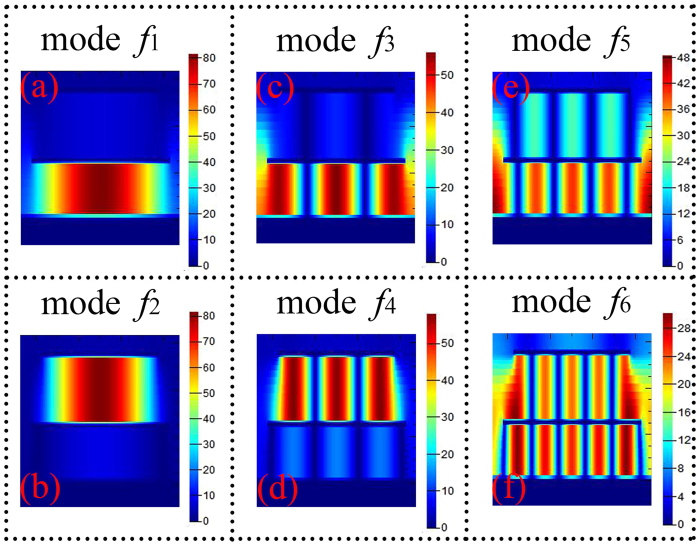
(**a**–**f**) are the magnetic field (|*H*y|, in the plane of *y* = 0) distributions of the six-band MPA at modes *f*_1_, *f*_2_, *f*_3_, *f*_4_, *f*_5_, and *f*_6_, respectively.

The resonance modes *f*_1_, *f*_3_, and *f*_5_ are the fundamental mode (i.e., the 1-order), 3-order, and 5-order responses of the metallic patch *l*_2_, respectively, while the modes *f*_2_, *f*_4_, and *f*_6_ are the 1-order, 3-order, and 5-order responses of the metallic patch *l*_1_, respectively. That is to say, the resonance modes of the *f*_1_, *f*_3_, *f*_5_ and *f*_2_, *f*_4_, *f*_6_ are the multiple-order responses of the metallic patches *l*_2_, and *l*_1_, respectively. Utilizing the classical antenna theory and *LC* resonance model, a further understand of the absorption mechanism of six-band MPA can be given^54^,^55^,^56^,^57^. This is a known fact that the operating frequency of the absorber satisfies the following form^54^,^55^,^56^,^57^:


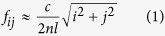


where *c* represents the light speed, *n* is the refractive index of the dielectric slab, *l* is the metallic pattern length, *i, j* = 0, 1, 2, … are integers. According to this equation, it is obvious that the operating frequency *f*_*ij*_ and the metallic pattern length *l* are inversely related. In particular, the operating frequencies of the 5-order (*i* = 5, *j* = 0) and 3-order (*i* = 3, *j* = 0) responses of the absorber should be about five and three times of the 1-order (*i* = 1, *j* = 0) response. In the six-band MPA, the frequencies of the modes *f*_5_ (2.75 THz) and *f*_3_ (1.74 THz) are about five and three times of the modes *f*_1_ (0.60 THz), respectively. The frequencies of modes *f*_6_ (3.63 THz) and *f*_4_ (2.33 THz) are about five and three times of the modes *f*_2_ (0.80 THz), respectively. Consequently, we can conclude that the resonance modes *f*_1_ (*f*_2_), *f*_3_ (*f*_4_), and *f*_5_ (*f*_6_) are indeed the 1-order, 3-order, and 5-order responses (or multiple-order responses) of the metallic patch *l*_2_ (*l*_1_), respectively. The slight frequency deviations are mainly derived from the interactions of these two metallic layers, see the field distributions in below Fig. 2.

The absorption mechanism (or physical origin) of the six-band MPA can be better gained by analyzing and investigating the distributions of the magnetic (|*H*y|) fields in six different resonance modes. As shown in Fig. 2, it is obvious that the |*H*y| distributions of the resonance modes *f*_1_, *f*_2_, *f*_3_, *f*_4_, *f*_5_, and *f*_6_ are primarily gathered in the dielectric layers of the six-band MPA. Thus, these resonance modes are all attributed to the localized EM responses of the MPA. In addition, we found that the field distributions of the modes *f*_1_, *f*_3_, and *f*_5_ are mainly focused on the dielectric layer *t*_2_, and the field distributions of modes *f*_2_, *f*_4_, and *f*_6_ are primarily gathered in the dielectric layer *t*_1_. These field distribution characteristics show that the modes *f*_1_, *f*_3_, and *f*_5_ are mainly associated with the excitation of the second metallic layer, while the modes *f*_2_, *f*_4_, and *f*_6_ are primarily the consequence of the excitation of the first metallic layer. To study the resonance mechanism of each resonance mode, we analyze the field distribution of each resonance mode in detail. The field distribution of the frequency at 0.60 THz (mode *f*_1_) is mainly distributed in the dielectric layer *t*_2_, while its field distribution at dielectric layer *t*_1_ is neglected, see Fig. 2(a). Besides, it is also found that there is only a resonance region (or a strong node) in the dielectric layer *t*_2_. Thus, the resonance mode *f*_1_ is the fundamental mode resonance (or 1-oder response) of the metallic patch *l*_2_. For resonance mode *f*_2_, its field distribution is primarily focused on the dielectric layer *t*_1_, see Fig. 2(b). We also observed that the field distribution of the mode *f*_2_ in dielectric layer *t*_1_ has only a strong node. As a result, the mode *f*_2_ is the 1-order response of the metallic patch *l*_1_. For resonance mode *f*_3_, three strong resonance regions are found in the dielectric layer *t*_2_, see Fig. 2(c). Similarly, three strong nodes are also observed in the dielectric layer *t*_1_ for resonance mode *f*_4_, see Fig. 2(d). Therefore, the resonance modes *f*_3_, and *f*_4_ are due to the 3-order response of the metallic patches *l*_2_, and *l*_1_, respectively. For resonance mode *f*_5_, see Fig. 2(e), its magnetic field is mainly gathered in the dielectric layer *t*_2_, while only a small part of magnetic field is observed in the dielectric layer *t*_1_. Besides, for field distribution of the resonance mode *f*_5_, it is noted that five strong nodes are found in the dielectric layer *t*_2_. Therefore, the resonance mode *f*_5_ is the 5-order response of the metallic patches *l*_2_. However, the field distribution of the mode *f*_6_ is different from the mode *f*_5_. As shown in Fig. 2(f), not only the dielectric layer *t*_1_ but also the dielectric layer *t*_2_ has strong field distributions for resonance mode *f*_6_. Notably, five strong resonance regions (or nodes) are found in both of the dielectric layers *t*_1_ and *t*_2_. Therefore, the resonance mode *f*_6_ is attributed to the hybridization (or coupling) of 5-order response of the metallic layers *l*_1_ and *l*_2_. Based on the combination of two sets of multiple-order responses (i.e., the 1-oder, 3-oder, and 5-order responses) of the two different sized metallic layers, six-band MPA is realized. The absorption mechanism of six-band MPA is different from previous multiple-band MPAs that only using the overlapping of the single resonance mode (in particular of the 1-order response) of metallic patterns^27^,^28^,^29^,^34^,^35^,^36^,^37^,^38^,^39^,^40^.

**Figure 3:**
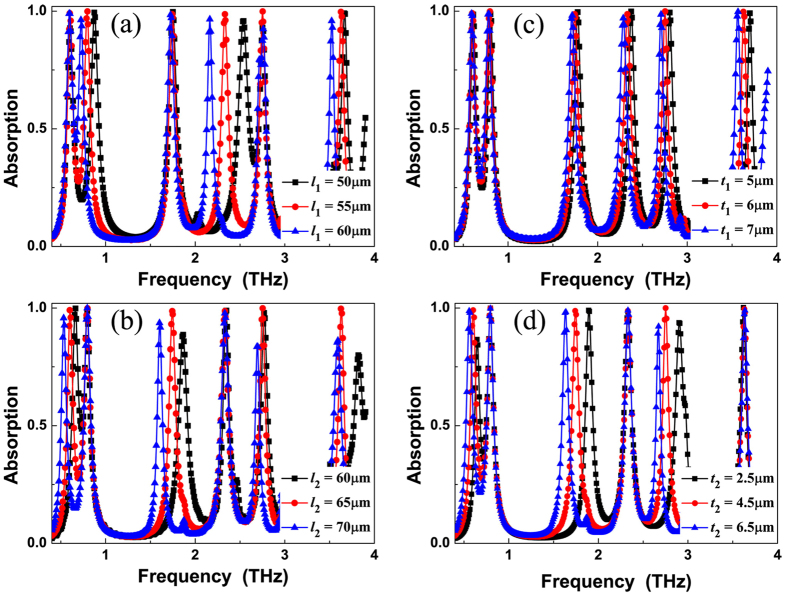
(**a**) is the dependence of the absorption spectra on the length change of the *l*_1_ (then *l*_2_ = 65 μm, *t*_1_ = 6 μm, and *t*_2_ = 4.5 μm); (**b**) shows the dependence of the absorption on the change of the *l*_2_ (then *l*_1_ = 55 μm, *t*_1_ = 6 μm, and *t*_2_ = 4.5 μm); (**c**) shows the dependence of the absorption on the thickness change of the *t*_1_ (then *l*_1_ = 55 μm, *l*_2_ = 65 μm, and *t*_2_ = 4.5 μm); (**d**) is the dependence of the absorption on the change of the *t*_2_ (then *l*_1_ = 55 μm, *l*_2_ = 65 μm, and *t*_1_ = 6 μm).

Because the resonance modes *f*_1_, *f*_3_, *f*_5_, and *f*_2_, *f*_4_, *f*_6_ are the multiple-order responses (1-order, 3-order, and 5-order resonances) of the metallic layer *l*_2_, and *l*_1_, respectively, the size change of the metallic layers will cause the shift of the frequencies in its corresponding resonance modes. As shown in Fig. 3(a), it is clear that the size change of *l*_1_ only affects the frequencies of the modes *f*_2_, *f*_4_, and *f*_6_, while the frequency changes of the modes *f*_1_, *f*_3_, and *f*_5_ are neglected. For change of the *l*_2_, see Fig. 3(b), the frequencies of the modes *f*_1_, *f*_3_, and *f*_5_ gradually decrease with the increase of the patch length *l*_2_. In addition, we also observed that the resonance frequency of the mode *f*_6_ has a large dependence on the change of the length *l*_2_. The results obtained in Fig. 3(a) and (b) are in agreement with the theoretical predictions. The adjustment or change of the resonance frequencies can also be obtained through varying the thicknesses of the dielectric layers *t*_1_, and *t*_2_. It can be seen from Fig. 3(c) that the change of the thickness *t*_1_ has a great influence on the resonance frequencies of the last four resonance modes (*f*_3_, *f*_4_, *f*_5_, and *f*_6_), while the frequency changes of the first two resonance modes are neglected. Moreover, for change of the thickness *t*_2_, see Fig. 3(d), it is found that its thickness change only influences the resonance frequencies of the modes *f*_1_, *f*_3_, and *f*_5_, while the frequencies of other resonance modes are nearly unchanged. Therefore, we can conclude that the size changes of the metallic layers and the thickness changes of the dielectric layers have the ability to adjust or shift the operating frequencies of the six-band MPA.

**Figure 4:**
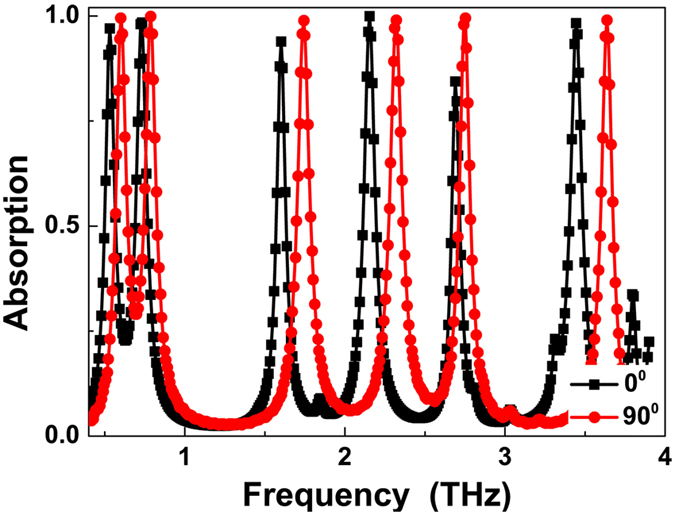
Absorption spectra of the six-band polarization tunable MPA.

Due to four-fold symmetric of these two metallic square patches, the six-band MPA presented here is polarization insensitive to the incident EM waves. Although the polarization insensitive six-band MPA has great application prospects, the development of the polarization tunable six-band MPA in many areas (such as specific EM wave polarization detection) is necessary and useful. Based on the results obtained in Fig. 3(a) and (b), in this paragraph, a six-band polarization tunable MPA through varying the sizes of the metallic layers in two orthogonal polarization directions is demonstrated. The sizes (in μm) of the presented six-band polarization tunable MPA are: *l*_1x_ = 60, *l*_2x_ = 70, *l*_1y_ = 55, *l*_2y_ = 65. The other parameters of the polarization tunable MPA are the same as those in Fig. 1(a) and (b). As illustrated in Fig. 4, six clear resonance bands are achieved at frequencies of 0.53 THz, 0.73 THz, 1.60 THz, 2.15 THz, 2.69 THz, and 3.44 THz with the average absorptivities greater than 95.29% in the 0 degree of polarization (i.e., along the *x*-axis). Six different absorption peaks are also observed in the polarization angle of 90 degrees (parallel to the *y*-axis). However, the resonance frequencies of the six-band MPA in the 90 degrees of polarization shift to 0.60 THz, 0.79 THz, 1.74 THz, 2.32 THz, 2.75 THz, and 3.64 THz, respectively. The frequency changes of the six resonance modes in 0 and 90 degrees are due to the different metallic layer sizes in two perpendicular directions. The six-band polarization tunable MPA can be potentially used to detect the EM waves of specific polarization and even to control and manipulate the polarization angle of incident waves^58^,^59^.

**Figure 5:**
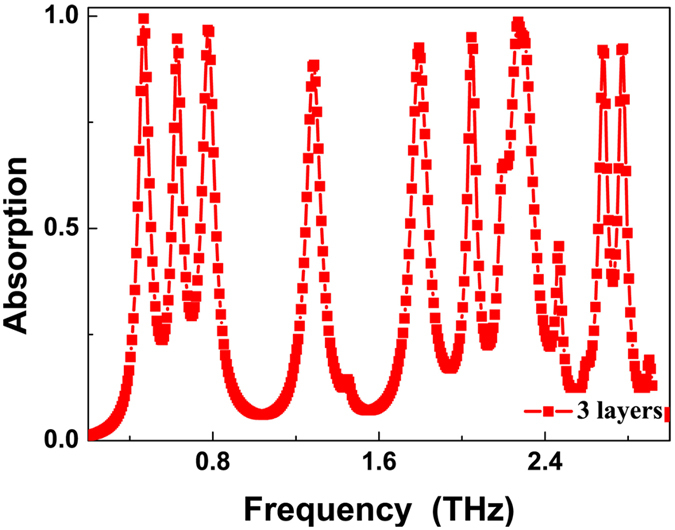
Simulated absorption spectra of the presented nine-band MPA (or three-layer stacked resonance structure).

The number of the resonance absorption peaks can be further increased by stacking more metallic layers. We use the three-layer stacked structure as an example to investigate its resonance absorption peaks. The optimal sizes (in μm, from top layer to bottom layer) of the presented three-layer stacked resonance structure are: *l*_1_ = 55, *l*_2_ = 65, *l*_3_ = 80, *t*_1_ = 7.6, *t*_2_ = 5.0, *t*_3_ = 6.1, *P* = 95. The other geometric parameters, including the refractive index of the dielectric layer and the metallic layer conductivity are the same as used in six-band MPA. The resonance absorption of the designed three-layer stacked MPA is illustrated in Fig. 5. Nine distinct absorption bands are obtained at frequencies of 0.47 THz (*f*_1_), 0.63 THz (*f*_2_), 0.78 THz (*f*_3_), 1.28 THz (*f*_4_), 1.79 THz (*f*_5_), 2.05 THz (*f*_6_), 2.27 THz (*f*_7_), 2.68 THz (*f*_8_), and 2.78 THz (*f*_9_) with the absorption of 99.45%, 94.77%, 96.75%, 88.55%, 92.60%, 95.04%, 98.68%, 92.03%, and 92.06%, respectively. The bandwidths of these peaks from low- to high-frequency are 0.067 THz, 0.060 THz, 0.077 THz, 0.088 THz, 0.095 THz, 0.056 THz, 0.049 THz, and 0.049 THz, respectively, and the *Q* values of nine resonance bands are 7.01, 10.50, 10.13, 14.55, 18.84, 36.61, 12.61, 54.69, and 56.73, respectively.

**Figure 6:**
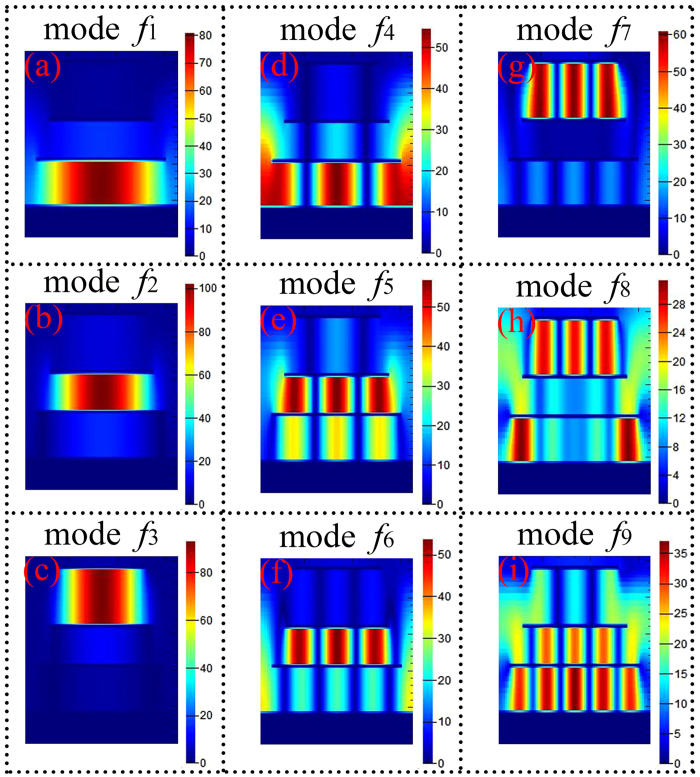
(**a**–**i**) Show the magnetic field (|*H*y|) distributions of the nine-band MPA at resonance modes *f*_1_, *f*_2_, *f*_3_, *f*_4_, *f*_5_, *f*_6_, *f*_7_, *f*_8_ and *f*_9_, respectively.

Furthermore, the distributions of the |*H*y| fields are provided to reveal or gain the absorption mechanism of the nine-band MPA, as shown in Fig. 6. Based on the above discussions and the |*H*y| field distributions of the different resonance modes in the nine-band MPA, we can conclude that the modes *f*_1_, *f*_2_, and *f*_3_ are the 1-order response of the metallic patches *l*_3_, *l*_2_, and *l*_1_, respectively, see Fig. 6(a–c). The resonance modes *f*_4_, and *f*_7_ are the 3-order response of the patches *l*_3_, and *l*_1_, respectively, see Fig. 6(d) and (g). The other resonance modes (*f*_5_, *f*_6_, *f*_8_, and *f*_9_) of the nine-band MPA are due to the hybridization of multiple-order responses of the metallic layers, see Fig. 6(e,f,h and i). For example, the resonance mode *f*_5_ originates from the hybridization (or coupling) of the 3-order responses of the metallic patches *l*_2_ and *l*_3_, see Fig. 6(e), and the mode *f*_6_ is derived from the coupling of the 3-order response of the metallic patch *l*_2_ and 5-order response of the metallic structure *l*_3_, see Fig. 6(f).

## Conclusion

In conclusion, a six-band polarization insensitive terahertz MPA based on a dual-layer stacked resonance structure is numerically investigated and studied. Six resonance bands with absorption average greater than 99.37% are achieved. Each layer of the resonance structure corresponds to three absorption peaks (1-oder, 3-order, and 5-order responses), and thus the absorption mechanism of the six-band MPA is attributed to the combination of two sets of the three resonance modes (1-oder, 3-order, and 5-order responses) of the two metallic layers in the stacked structure. The field distributions of the six resonance bands are given to reveal or gain the absorption mechanism of the six-band MPA. Besides, the design also has the ability to tune or change the resonance frequencies of the six-band MPA through varying the sizes of the metallic layers or the thicknesses of the dielectric layers. Employing this results, a six-band polarization tunable MPA is also analyzed and demonstrated. Furthermore, the number of the resonance bands or peaks can be further increased by stacking more metallic square patches. As an example, we found that three-layer stacked resonance structure can achieve nine-band near-perfect absorption. Six-band and even nine-band MPAs obtained here can find potential applications in many optoelectronic related areas. Once the structure is fabricated, however, the absorption performance (including the resonance frequency and the absorption strength) of the absorber is unable to change. Some temperature-dependent materials, bias-tunable materials, and even microfluidic systems can be used to overcome this kind of shortcoming. The further works are the design of the active tunable multiple-band light absorbers.

## Methods

### Numerical modeling and detailed parameters of the presented MPA

The geometric parameters of the six-band MPA are shown in Fig. 1(a) and (b). Detailed parameters (in μm) of the six-band MPA are: *l*_1_* = l*_1x_ = *l*_1y_ = 55, *l*_2_* = l*_2x_ = *l*_2y_ = 65, *t*_1_ = 6, *t*_2_ = 4.5, *P* = *Px* = *Py* = 75. The metallic layers of the six-band MPA are made of Au, and the thickness and conductivity of them are 0.4 μm, and 4.09 × 10^7^ S/m, respectively. The refractive index of the dielectric layer is *n* = 3.2 + *i*0.025. In this investigation, we utilize normally incident EM waves with *E* field parallel to the *x*-axis as illustrated in Fig. 1(b). Periodic boundary conditions are employed along the direction of the wave propagating, and in the normal to the wave propagating direction, perfect matching layers are used. The frequency dependent absorption can be given by: *A*(w) = 1 − *T*(w) − *R*(w), where *T*(w) and *R*(w) are the frequency dependent transmission and reflection, respectively. *T*(w) is close to zero because of the presence of the metallic board. As a result, the *A*(w) can be obtained using *A*(w) = 1 − *R*(w). We can achieve perfect absorption (*A*(w) = 1) when the *R*(w) is completely suppressed (i.e., the impedance of the structure is equal to that of the air).

## Additional Information

**How to cite this article**: Wang, B.-X. *et al*. Six-band terahertz metamaterial absorber based on the combination of multiple-order responses of metallic patches in a dual-layer stacked resonance structure. *Sci. Rep.* **7**, 41373; doi: 10.1038/srep41373 (2017).

**Publisher's note:** Springer Nature remains neutral with regard to jurisdictional claims in published maps and institutional affiliations.
